# Improving immersive experiences in virtual natural setting for public health and environmental design: A systematic review and meta-analysis of randomized controlled trials

**DOI:** 10.1371/journal.pone.0297986

**Published:** 2024-04-17

**Authors:** Yuhan Wen, Xiwei Shen, Yan Shen

**Affiliations:** 1 Yueyang Hospital of Integrated Chinese and Western Medicine, Shanghai University of Traditional Chinese Medicine, Hongkon District, Shanghai, China; 2 School of Architecture, Tianjin University, Nankai District, Tianjin, China; 3 School of Architecture, University of Nevada, Las Vegas, Nevada, United States of America; Queen’s University Belfast, UNITED KINGDOM

## Abstract

In recent years, virtual reality (VR) technology has emerged as a powerful tool in the field of therapeutic landscapes. For hospitalized patients or individuals with limited mobility, VR provides highly personalized therapy by simulating authentic natural environments within a safe, convenient, and engaging setting. This study investigated the effectiveness of immersing patients in virtual natural environments for health recovery and compared the varying impacts of different types of landscapes on patients’ recovery levels. The aim was to complement traditional medical approaches and enhance environmental design in the field of public health. Researchers systematically reviewed databases (January 2018 to August 2, 2023) to identify randomized controlled trials comparing the efficacy of virtual nature immersion with other treatments. The inclusion/exclusion criteria were established based on the population, intervention, comparison, outcomes, study design, and other aspects (expanded PICO) framework. The Cochrane tool was employed to assess the risk of bias. Meta-analysis was conducted by pooling the mean differences with a 95% confidence interval. Among 30 trials, a total of 2123 patients met the inclusion criteria, with 15 studies included in the meta-analysis. 30 trials met the criteria. Results show significant improvements in pain, anxiety, fear, and some physiological indicators with virtual nature-based treatments. On the other hand, natural scenes incorporating blue and green elements have been applied more extensively and have shown more significant effects. In comparison to conventional methods, this study strongly advocates that virtual reality environments are a crucial tool in bridging the gap between patients and nature, demonstrating their potential to reshape medical interventions and improve environmental design in the field of public health.

## Introduction

Nature has long been recognized for its profound impact on human health and well-being. The therapeutic benefits of exposure to natural environments, often referred to as "nature therapy," are well-established [1]. In terms of mental wellness, immersing oneself in nature can improve attention, increase positive emotions, enhance life satisfaction, and reduce depression, stress, and anxiety [2–4]. Physiologically, the unique natural sights and sounds of green spaces can alleviate pain perception [5], increase physical activity levels, and reduce the incidence of cardiovascular diseases and obesity [6]. Additionally, natural environments encourage increased social interaction, strengthening social support networks [7]. These findings underscore the potential of integrating natural elements into medical treatment and rehabilitation plans, particularly in the context of high stress, anxiety, and fast-paced modern societies.

However, integrating patients with natural environments can be challenging due to factors such as physical limitations, medical equipment, and confined spaces. This limitation can exacerbate psychological symptoms, intensifying fear, anxiety, and depression associated with their conditions. Yan (2020) demonstrated that patients who are confined to hospitals for extended periods due to chronic health issues experience lower levels of quality of life and heightened psychological stress [8]. Ferreira (2021) assessed that individuals under mandatory home quarantine during the COVID-19 pandemic generally exhibited higher levels of anxiety and lower quality of life compared to pre-quarantine levels [9]. Consequently, the importance of nature for patients is increasingly recognized, leading to the implementation of measures such as creating healing gardens [10], optimizing scenic views from windows [4], harnessing natural light [11], and employing virtual reality technology [12] to offer patients more immersive natural experiences.

In recent years, propelled by remarkable advancements in computer technology and graphic processing capabilities, virtual reality (VR) technology has emerged as a powerful tool within the realm of healing and therapeutic landscapes. VR relies on sophisticated computer systems to foster immersive experiences by creating intricate three-dimensional environments or scenarios [13]. Through VR, healthcare professionals and designers can craft highly realistic and personalized environments such as forests, oceans, or mountains. VR also allows for the customization of therapeutic environments, catering to specific needs and health conditions of patients, ranging from vibrant wilderness biking in forests to serene lakesides or coastlines [14, 15]. Additionally, VR technology can provide patients with interactivity and a sense of engagement. By utilizing devices like head-mounted displays, patients can interact with virtual environments, engaging in activities such as strolling along a beach or frolicking amidst fish and corals in an underwater world [16, 17]. This interactivity contributes to enhancing the emotional state and cognitive abilities of patients.

In clinical treatment, virtual natural scenes constructed by VR can ameliorate various physiological or psychological symptoms in patients. For example, utilizing VR to simulate natural settings during medical procedures has shown potential in alleviating pain and reducing anxiety [18]. Similarly, Eraslan Boz (2020) used virtual natural environments to assess cognitive function in elderly individuals, demonstrating its potential in enhancing cognitive well-being [19]. By leveraging VR technology, designers can create virtual landscapes that offer a sense of tranquility and facilitate healing, holding potential benefits for improving mental health, managing pain, promoting recovery, and enhancing overall well-being [20–22].

Currently, healthcare practitioners and researchers are increasingly exploring the potential of utilizing VR in therapeutic environments to enhance conventional treatments. However, due to factors such as limited research participants, variations in study populations, and discrepancies in settings, individual studies often suffer from a scarcity of research subjects and conflicting conclusions. To comprehensively understand the efficacy of immersing patients in VR-generated natural environments for recovery, there is a need for a systematic review of existing randomized controlled trials (RCTs). This paper conducts a systematic review and meta-analysis of RCTs conducted in the last five years, examining the characteristics and objectives of patient populations involved in existing trials. It consolidates virtual immersion in natural environments, compiles and categorizes virtual natural scenes, and assesses the efficacy of immersing patients in virtual nature across various aspects of patient health and healing.

## Methods

### 1 Eligibility criteria

This systematic review follows the Preferred Reporting Items for Systematic Reviews and Meta-Analyses (PRISMA) guidelines [23]. The inclusion/exclusion criteria were determined based on the Population, Intervention, Comparison, Outcome, Study Design and Others (Extended PICO) framework (Table 1). The primary objective of this review is to assess the effectiveness of virtual immersion in nature promoting patient psychological well-being, physiological effects, pain management, as well as patient preferences and satisfaction. Examining the impact of virtual nature experiences on patients’ mental and emotional well-being, which may involve measuring factors like reduced stress levels, improved mood, and decreased anxiety or depression symptoms. Furthermore, investigating any physiological effects of virtual nature immersion, such as changes in heart rate, blood pressure, or cortisol levels. These indicators can provide insight into the potential stress-reducing properties of the intervention. Finally, Gathering feedback from patients about their preferences for virtual nature experiences and their overall satisfaction with this intervention as part of their recovery process.

**Table 1:** Extended PICO for this review.

Review Questions	Inclusion Criteria	Exclusion Criteria
Population	Patients	
Intervention	Natural environments or highly realistic computer-generated natural scenes; Immersive or semi-immersive VR methods	Unknown or Non-natural scenes; Non-immersive VR methods
Comparator	Any comparator	
Outcomes	Physical or psychological outcome	Other outcomes (e.g., limb movement training, cognitive training)
Study Design	Randomized controlled trials	Not randomized controlled trials
Others	Published in a peer-reviewed academic journal in English; January 2018 to July 2023	

Inclusion criteria encompass: (1) studies designed as randomized controlled trials; (2) interventions in the experimental group must involve natural environments or highly realistic computer-generated natural scenes, such as parks, beaches, forests, etc.; (3) study subjects are patients, with no age restrictions; (4) interventions involve immersive or semi-immersive VR methods; (5) studies published between January 2018 and July 2023; (6) articles are in English; (7) study results must include physical or psychological outcome indicators during the treatment process.

Exclusion criteria include: (1) study designs that are not randomized controlled trials, including single-arm trials, observational studies, reviews or descriptive articles, study protocols, qualitative studies, case reports, and grey literature; (2) interventions involving non-natural scenes, and studies that do not differentiate between natural and non-natural scenes, such as indoor settings, urban streets, stadiums, cartoon or game environments, etc.; (3) interventions utilizing non-immersive VR methods; (4) primary purposes of the study unrelated to the intervention setting, such as limb movement training, cognitive training, etc.

### 2 Information sources and search strategy

Literature was identified by searching the following databases: Medline, Embase, Scopus, Pubmed, and Web of Science. The search was conducted by two independent researchers (W and S) and was completed on August 2, 2023. The search strategy is outlined in Table 2. Keywords were extracted from previous works by Frost (2022) [20] and Ohly (2016) [24]. Search results were restricted to English language papers/RCTs published from 2018 to July 2023.

**Table 2:** Key word combinations used (adapted for each database).

Title must include	Virtual
Abstract must include	patient* OR outpatient* OR inpatient*
Abstract must include	setting* OR environ* OR marine OR forest OR sea OR ocean OR natur* OR wilderness OR park* OR open space* OR green space OR greenspace OR wood* OR bush OR countryside OR outdoor* OR grassland OR coastal OR green OR garden* OR landscape* OR design OR condition*

Note. Search terms were combined into one search.

### 3 Selection process

All identified references from the search strategy were uploaded to ENDNOTE, and duplicate references were removed. Two reviewers (W and S) independently screened titles and abstracts. Reasons for exclusion at the full-text screening stage were primarily due to interventions in non-natural settings (e.g., supermarkets or game scenes) or study populations (e.g., healthy individuals or including doctors and nurses). Disagreements were resolved through discussion.

### 4 Data extraction

A standardized data extraction form was developed in Excel. Data extracted for each study included sample characteristics, intervention type, category of nature, outcome measures, and study results. Data were extracted by one reviewer (W) independently and checked by another reviewer (S). Disagreements were resolved through discussion, involving the entire team if necessary.

### 5 Study risk of bias assessment

Two reviewers (W and S) independently assessed the methodological quality and risk of bias of included studies using Review Manager 5.4 software (Cochrane Library, http://www.cochranelibrary.com/) based on the Cochrane Handbook for Systematic Reviews of Interventions (http://www.cochranelibrary.com/). This handbook comprises seven domains: (1) random sequence generation, (2) allocation concealment, (3) blinding of participants and personnel, (4) blinding of outcome assessment, (5) incomplete outcome data, (6) selective reporting, and (7) other bias. Disagreements were resolved through discussion. Each domain was judged as "low risk of bias," "high risk of bias," or "unclear risk of bias" based on the bias risk assessment criteria.

### 6 Statistical analysis

Meta-analysis was conducted using Review Manager 5.4 software. For continuous variables, inverse variance was used to pool mean differences (MDs) with 95% confidence intervals (CIs). Heterogeneity between studies was assessed using the Q-test and I^2^ statistic. Interpretation of I^2^ statistics followed the Cochrane Handbook for Systematic Reviews of Interventions. If the Cochrane Q statistic had a p-value < 0.10 and I^2^ statistic was > 50%, significant heterogeneity was considered present, and a random-effects model was employed. Conversely, a fixed-effects model was used [25].

## Results

### 1 Study selection

**Fig 1:**
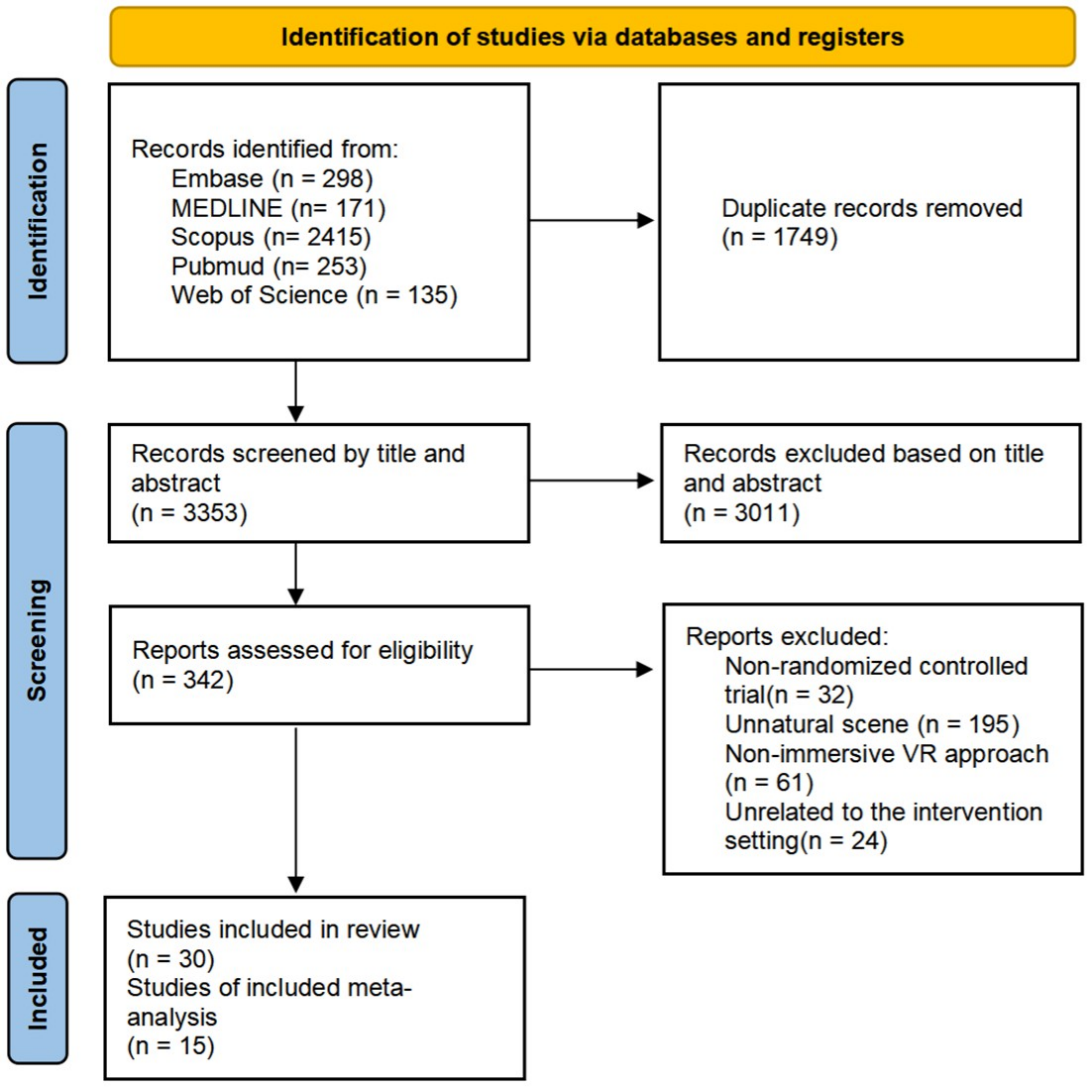
PRISMA flow diagram.

Fig 1 illustrates the PRISMA flowchart for this study. A total of 5102 records were retrieved from five databases. After removing duplicates using Endnote, a preliminary screening resulted in 3353 articles. Two authors (W and S) independently screened the titles and abstracts of these articles according to the inclusion criteria, resulting in the exclusion of 3011 articles in this stage. Subsequently, 342 full-text articles were independently assessed for eligibility. Based on the exclusion criteria, 312 articles were excluded for various reasons, including interventions in non-natural settings such as game, indoor, or cartoon scenes; interventions utilizing non-immersive VR technology; and study populations not consisting of patients. Finally, 30 randomized controlled trials were selected for qualitative and quantitative synthesis.

### 2 Virtual natural environment interventions

**Fig 2:**
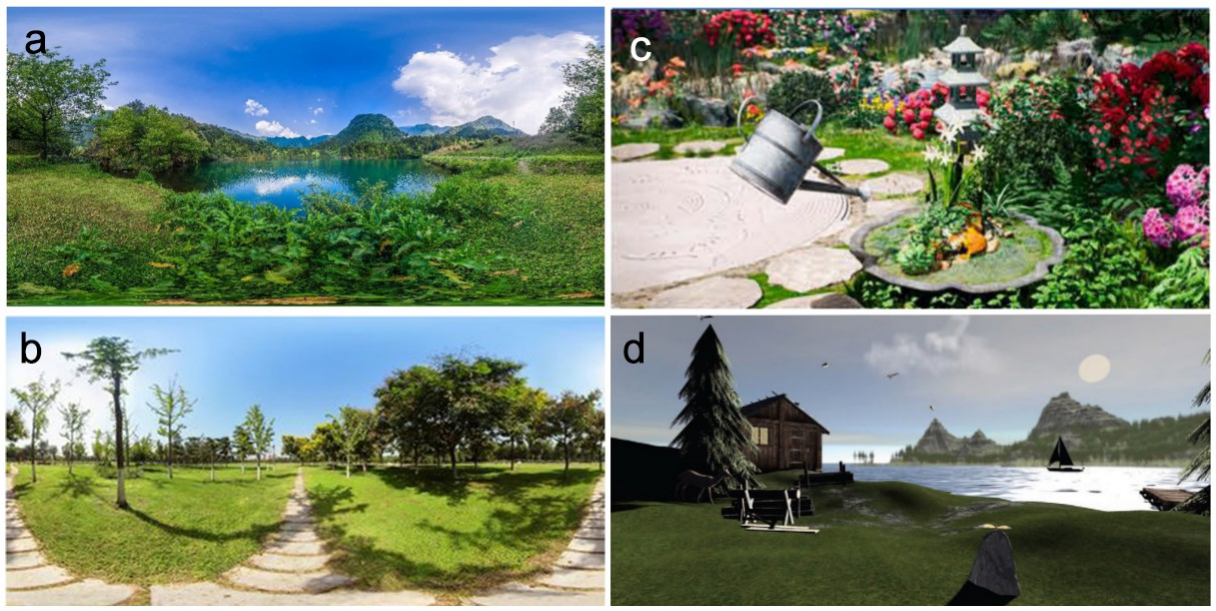
Contrast of nature scenes in VR. (a, b) Real natural scene. (c, d) Highly simulated natural scenes. (a)Screenshot of the virtual environment in the biophilic VR condition [30]. Source: Luo, W., Chen, C., Zhou, W., Cao, A., Zhu, W., Zhou, Y.,… & Zhu, B. (2023). Biophilic virtual reality on children’s anxiety and pain during circumcision: A randomized controlled study. Journal of pediatric urology, 19(2), 201–210. (b)Semi-open green space [29]. Source: Song, R., Chen, Q., Zhang, Y., Jia, Q. A., He, H., Gao, T., & Qiu, L. (2022). Psychophysiological restorative potential in cancer patients by virtual reality (VR)-based perception of natural environment. Frontiers in Psychology, 13, 1003497. (c)The virtual garden [31]. Kiper, P., Przysiężna, E., Cieślik, B., Broniec-Siekaniec, K., Kucińska, A., Szczygieł, J.,… & Szczepańska-Gieracha, J. (2022). Effects of immersive virtual therapy as a method supporting recovery of depressive symptoms in post-stroke rehabilitation: Randomized controlled trial. Clinical Interventions in Aging, 1673–1685. (d)Virtual reality and virtual reality hypnosis landscape designed by Oncomfort [32]. Rousseaux, F., Dardenne, N., Massion, P. B., Ledoux, D., Bicego, A., Donneau, A. F.,… & Vanhaudenhuyse, A. (2022). Virtual reality and hypnosis for anxiety and pain management in intensive care units: a prospective randomised trial among cardiac surgery patients. European journal of anaesthesiology, 39(1), 58.

**Fig 3:**
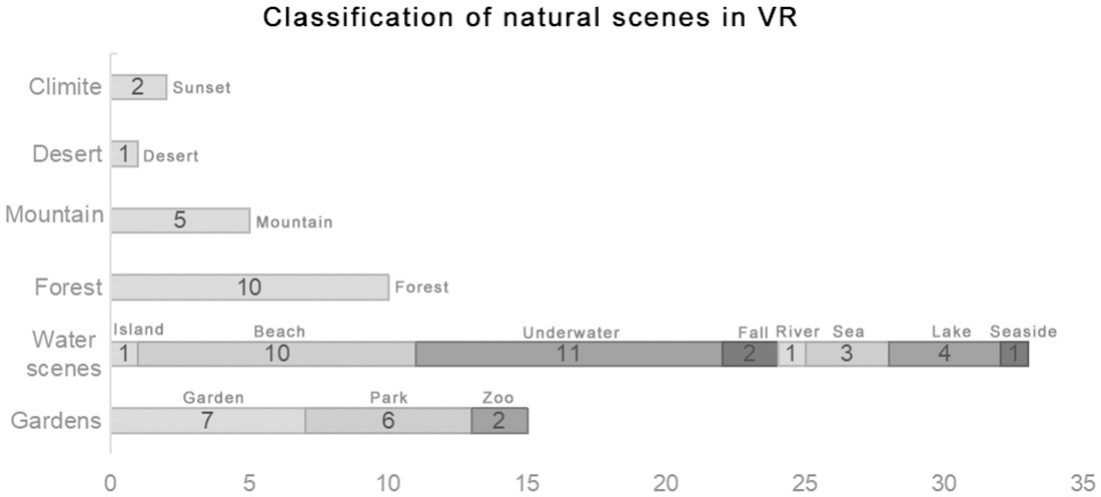
Classification of natural scenes in VR. The different intervention types within the same article were counted multiple times.

All experimental group patients wore VR goggles during the intervention, and in most cases, headphones were provided to play relaxing music. The virtual natural scenes used in the interventions of these studies were aggregated, but due to some experiments employing multiple scenes, some studies were counted more than once in the statistics. These research scenarios can be categorized into real natural scenes and highly realistic simulated natural scenes (Fig 2). Among these natural scenes, the most frequently occurring were aquatic landscapes, totaling 38, including islands, seascapes, beaches, lakesides, riversides, waterfalls, and 11 underwater seascapes. There were 14 instances of garden or park landscapes. Eleven studies utilized natural forest or woodland landscapes. Five studies featured mountain landscapes, and one included a desert landscape (Fig 3). In the control groups, most interventions involved either not using VR goggles and receiving standard care or, in some cases, providing analgesics or sedatives. In one experiment [26], participants used an iPad to view real-world scenes in the VR device. In another experiment [27], standard care was provided in the first week, followed by a VR program in the second week. In another study [28], patients watched watercolor paintings while engaging in indoor cycling. Additionally, a different experiment [29] utilized VR devices to view indoor grayscale spaces.

### 3 Study characteristics

Table 3 summarizes the characteristics of these studies. The sample comprises 30 peer-reviewed studies, including a total of 2123 patients. The studies primarily originated from Asia (n = 13), Europe (n = 11), North America (n = 4), and Australia (n = 2). Of the 30 studies, 18 focused on adult patients undergoing surgery or clinical treatment, who were likely to experience depression, anxiety, and pain. Seven studies exclusively targeted adult females undergoing surgery or clinical examinations, with one specifically addressing elderly females with depression or anxiety disorders. Five studies aimed at preoperative or intraoperative pain or anxiety relief in children or adolescents. Sample sizes ranged from 15 to 255 patients. One experiment employed a crossover trial design, involving one patient [33].

**Table 3:** Basic information of included studies.

Study	Participants Characteristics Mean age (years)	Intervention		Type of nature	Control group	Main outcome Measures	Significant outcomes by type
		Sample size (Male/ Female)	Experimental group				
Sewell, 2023 [34]	Women underwent OPH E = 47.8±11.3 C = 49.6±11.3	83(0/83) E = 41 C = 42	VR training OPH procedure	Japanese zen garden Relaxing music	OPH procedure Paregoric	11-point NRS • Anxiety • Pain • Satisfaction	Reduce anxiety Not reduce pain and enhance satisfaction
Luo, 2023 [30]	Children during circumcision 10.8(8–14) E = 10.5(8–14) C1 = 11.0(8–14) C2 = 10.8(8–14)	106 E = 34 C1 = 36 C2 = 36	VR training	Rural scenery Soft music	C1: Using VR glasses to have an indoor apartment scene C2: not wear VR glasses	CmYPAS, VAS, HR, Ai. • Anxiety FPS-R, Pi • Pain	Reduce pain and anxiety
Cieslik, 2023 [33]	Elderly women with depressive 68.15±5.53 E = 68.77±5.57 C = 67.53±5.51	60(0/60) E = 30 C = 30	Eight sessions of VR training Eight sessions GFT	Japanese zen garden Relaxing music	Eight sessions of relaxation and psychoeducati-on Eight sessions GFT	GDS-30 • Depression HADS • Anxiety • Depression	Reduce depressive and anxiety
Rutkowski, 2023 [35]	Patients with COVID-19 57.8±4.92 E = 59.22±3.89 C = 56.00±6.57	32(12/20) E = 18(7/11) C = 14(5/9)	A bicycle ride with VR goggles	Sunny island Therapeutic garden Sound effects	Exercise training Schulz autogenic training	6MWT, FEV1, FVC and FEV1/VC, TLC • Functional capacity PSS-10 • Stress	Reduce stress, improve exercise performance Not lung function
Kim, 2023 [36]	Patients with ESD E = 56.65±8.62 C = 59.65±6.92	40(28/12) E = 20(13/7) C = 20(15/5)	VR training by VR meditation program	Gardens, beach Underwater scenes	No specific intervention	STAI • Anxiety VAS • Pain 5-point Likert scale • Satisfaction	Enhance overall satisfaction, reduce anxiety Not pain
Korkmaz, 2023 [37]	Adult patients with BMAB procedure E = 49.86±15.63 C = 50.18±16.26	75(40/35) E = 35(19/16) C = 40(21/19)	VR training	Underwater Park	No specific intervention	STAI • Anxiety VAS • Pain	Reduces pain and anxiety
Sezer, 2023 [38]	Women underwent HSG 30.97±27.40 E = 30.41±6.41 C = 31.52±5.58	62(0/62) E = 31 C = 31	VR training Relaxing music HSG	Sea Underwater Forest	HSG	SAI • Anxiety VAS • Pain • Fear • Satisfaction Vital signs	Reduce pain and fear Increase satisfaction Not affect anxiety and vital signs
Ugras, 2023 [38]	Patients underwent surgery E = 44.7±12.9 C = 43.0±15.8	86(59/27) E = 43(30/13) C = 43(29/14)	VR training	Underwater world Forest Park Beach Relaxing music	No specific intervention	ASSQ • Anxiety Vital signs	Reduce anxiety and physiological responses
Kiper, 2022 [31]	Patients with stroke E = 65.50±6.72 C = 65.57±4.99	60(30/30) E = 30(13/17) C = 30(17/13)	VR training Standard procedure	Therapeutic garden	Standard procedure	GDS-30 • Anxiety GSES • Self-efficacy AIS • Acceptance VAS • Pain HADS • Anxiety • Depression BI, IADL, RMA • Functional parameters	Reduce depressive and anxiety, improve mood Not functional aspects and pain
Song, 2022 [29]	Cancer patients, E = 61.31±15.10 C = 59.48±12.06	63(43/20) E = 32(23/9) C = 31(20/11)	VR training by virtual green and blue spaces	Grassland Woods Lake	VR training by virtual gray space	SDS • Depression PRS • Perceived restoration PANAS • Emotions Seven-point Likert scale • Perceived preferences Vital signs	Increase positive emotions and perceived restoration, decrease negative emotions and HR
Menekli, 2022 [39]	Cancer patients underwent port catheter implantation	139(52/87) E = 69(29/40) C = 70(23/47)	VR training	Parks Seaside, Submarine Relaxing music	No specific intervention	SAI • Anxiety VAS • Pain Vital signs	Decrease pain, anxiety and SBP, DBP, HR, and RR, increase SpO_2_
Rousseaux, 2022 [32]	Cardiac surgery patients 66±11.5 E1 = 64.7±13.4 E2 = 68.4±7.8 C1 = 63.3±11.5 C2 = 67.6±12.5	100(76/24) E1 = 25(19/6) E2 = 25(21/4) C1 = 25(18/7) C2 = 25(18/7)	E1 = A 20 min virtual reality session Daily standard care E2 = A 20 min virtual reality hypnosis session Daily standard care	A mountain cabin near a lake at sunrise	C1 = Daily standard care C2 = 20 min hypnosis session Daily standard care	VAS • Anxiety • Pain • Relaxation • Fatigue Vital signs Opioid use	No significant differences
Wang, 2022 [40]	Generalized anxiety disorder patients 59.86±7.46 E1 = 58.43±7.37 E2 = 59.87±6.99 C1 = 30±60.85	90(41/49) E1 = 30(13/17) E2 = 30(14/16) C = 30(14/16)	E1 = Cycle for 20 min in Cave VE through high tree density sportscape E2 = Cycle for 20 min in Cave VE through medium tree density sportscape	Forests Parks Trees Rivers	Cycle for 20 min	Pupil size • Stress Fixation count and time • Visual attention	High tree density sportscape get more visual attention Medium tree density sportscape reduce stress more
Melcer, 2021 [41]	Women undergoing mid-trimester amniocentesis E = 34.9±4.9 C = 36.7±3.3	60(0/60) E = 30 C = 30	VR training	Rolling hills Beach Desert, Undersea world	See the intervention on the ultrasound monitor.	VAS • Pain STAI • Anxiety	Reduce pain Not anxiety
Chang, 2021 [26]	Patients undergoing in-office laryngotracheal procedures 59.4±16.7 E = 56.6±16.4 C = 62.9±16.5	15(9/6) E = 8(4/4) C = 8(5/3)	VR training Local anesthesia	Beach	Local anesthesia	SUDS • Anxiety VAS • Pain 7-point Likert scale • Satisfaction scores Procedure time HADS • Baseline anxiety	Reduce anxiety Not reduce pain and procedure time and enhance satisfaction
Jozwik, 2021 [42]	Patients undergoing phase II CR 64.70±8.03 E = 64.70±8.03 C = 66.00±9.73	77(35/42) E = 28(11/17) C = 49(24/25)	Eight sessions of VR therapy	Therapeutic garden	Eight sessions of Schultz Autogenic Training	HADS • Depression • Anxiety PSQ • Stress	Reduce anxiety and depression
Karaman, 2021 [43]	Woman undergoing FNA breast biopsies E = 46.9±11.2 C = 41.4±12.9	60(0/60) E = 30 C = 30	VR training	Beach Relaxation music	No specific intervention	VAS • Pain STAI • Anxiety	Reduce pain and anxiety
Buyuk, 2021 [44]	Children during circumcision	78 E = 40 C = 38	VR training	Amazon forests Water skiing	Conventional mode of education	CFS • Fear CAMS • Anxiety WBS • Pain	Reduce anxiety, fear and pain
Gerçeke, 2021 [45]	Children or adolescents with cancer E = 6–12: 12±57.1; 13–17: 9±42.9 C = 6–12: 12±57.1; 13–17: 9±42.9	42(26/16) E = 21(13/8) C = 21(13/8)	VR training	Underwater Forest	No specific intervention	WBS • Pain CFS • Fear CAM-S • Anxiety	Reduce pain, fear and anxiety
Brewer, 2021 [46]	Patients undergoing an office-based endovenous radiofrequency ablation	40 E = 20 C = 20	VR training	Beach	No specific intervention	WBS • Anxiety	Reduce anxiety
Deo, 2021 [47]	Women underwent an outpatient hysteroscopy E = 31.1±5.4 C = 31.3±5.2	40(0/40) E = 20 C = 20	VR training	Rainforest Lake	A routine procedure	11-point NRS • Pain • Anxiety	Reduce pain and anxiety
Tennant, 2020 [48]	Oncology inpatients E = 11.59±3.61 C = 11.60±2.77	90(50/40) E = 61(37/24) C = 29(13/16)	VR training	Australian national parks Australian zoos	Using an iPad to deliver identical content presented in the VR condition	VAS • Child state • Positive mood • Anxiety • Anger • Nause • Pain SpO_2_ SCAS • Trait Anxiety ARI • Immersion	Increase positive mood and reduce negative symptoms
Lahti, 2020 [49]	Adult patients with dental treatment 52.5±16.4 E = 51.8±16.8 C = 53.3±16.0	255(84/171) E = 129 C = 126	VR training	Beach Waterfall Underwater Relaxing music	Remain seated for 3 min	MDAS • Anxiety	Reduce anxiety
Wong, 2020 [50]	Women in labor E = 32.5±3.6 C = 31.6±5.6	40(0/40) E = 21 C = 19	VR training	Blossoming tree Ocean waves	No specific intervention	MCID • pain BP HR	Reduce pain
Lakhani, 2020 [27]	People with a SCI E = 56.20±20.74 C = 48.00±16.21	24(16/8) E = 10(10/0) C = 14(6/8)	The first week: up to three 20-minute VR sessions over three consecutive days during one week The second week: regular rehabilitation practice over a week	Underwater Zoo Canyon Park Falls	The first week: regular rehabilitation practice over a week The second week: up to three 20-minute VR sessions over three consecutive days during one week	PHQ-8, feeling intensity scales • Depression • Anxiety	Improve mental health and reduce anxiety
Navarro-Haro, 2019 [51]	Patients with GAD 45.23±11.23 E = 45.05±8.17 C = 45.40±13.74	39(9/30) E = 19(4/15) C = 20(5/15)	VR training Seven MBI group sessions	Underwater	Seven MBI group sessions	GAD-7 • Anxiety HADS • Anxiety • Depression FFMQ • Mindfulness DERS • Emotion regulation MAIA • Interoceptive awareness ITC-SOPI • Experience VAS • Emotion SPQ • Presence	Treat GAD symptoms, depression, anxiety, and emotion dysregulation
Wang, 2019 [14]	Patients with GAD E = 44.27±10.25 C = 50.50±15.68	60(25/35) E = 30(12/18) C = 30(13/17)	A bicycle ride with VR goggles	Park road	A bicycle ride with observing watercolor painting images projected by the projector	GSR, HR, EEG • Stress	Higher exercise intensity and lower perceived emotional stress
Sharifpour, 2019 [52]	Adolescents with cancer E = 14.8±2.4 C = 15±1.85	30 E = 15 C = 15	Eight 30-min sessions of VRT once a week for 2 months	Sunset Beach, Underwater	No specific intervention	MPQ • Pain intensity PASS • Pain anxiety PCS • Pain catastrophizing PSEQ • Pain self-efficacy	Reduce pain anxiety, intensity, catastrophisi-ng, self-efficacy
McCune, 2023 [53]	Adult underwent a laparoscopic hysterectomy E = 41.7±9.5 C = 40.9±14.3	30(0/30) E = 15 C = 15	VR training IV pain medication Routine PACU	Lake Mountains Forest Waves Beach	IV pain medication Routine PACU	VAS • Pain	Well received by patients, Not improve pain scores or decrease narcotic usage
Larsson, 2023 [53]	Patients referred for ICA E = 62.5±10.9 C = 62.6±9.5	156(111/45) E = 80(55/25) C = 76(56/20)	VR training	Zen garden Forest Mountain Beach Diving	No specific intervention	SNDD, VASA • Anxiety	Not reduce anxiety

E, experimental group; C, control group; OPH, outpatient hysteroscopy; NRS, numerical rating scale; CmYPAS, Chinese version of the modified Yale Preoperative Anxiety Scale; VAS, Visual Analogue Scale; HR, heart rate; Ai, Anxiety index; FPS-R, the Faces Pain Scale-Revised; Pi, Pain index; GDS-30, 30-item Geriatric Depression Scale; HADS, Hospital Anxiety and Depression Scale; 6MWT, 6-minute walk test; FEV1, forced expiratory volume for 1 second; FVC, forced vital capacity; TLC, total lung capacity; PSS-10, The Perceived Stress Scale; ESD, endoscopic submucosa dissection; STAI, the Spielberger’s State-Trait Anxiety Inventory; BMAB, bone marrow aspiration and biopsy; HSG, Hysterosalpingography; ASSQ, the Anxiety Specific to Surgery Questionnaire; GSES, the Generalized Self-Efficacy Scale; AIS, the Acceptance of Illness Scale; BI, the Barthel Index; IADL, the Lawton Instrumental Activities of Daily Living Scale; RMA, the Rivermead Motor Assessment; SDS, the Self-Rating Depression Scale; PRS, Perceived Restorativeness Scale; PANAS, the Positive and Negative Affect Scale; SBP, diastolic blood pressure; DBP, diastolic blood pressure; RR, respiratory rate; SUDS, the Subjective Units of Distress Scale; CR, cardiac rehabilitation; PSQ, The Perception of Stress Questionnaire; FNA, fine needle aspiration; CFS, children’s fear scale; CAMS, children’s anxiety meter scale; WBS, the Wong-Baker faces pain rating scale; CFS, children’s fear scale; SCAS, Spence Children’s Anxiety Scale; the Augmented Reality Immersion; MDAS, Modified Dental Anxiety Scale; MCID, minimum clinically important differences; BP, blood pressure; PHQ-8, The Patient Health Questionnaire-8; SCI, Spinal Cord Injury; GAD, Generalized Anxiety Disorder; GAD-7, General anxiety disorder 7 items; FFMQ, Five facets of mindfulness questionnaire; DERS, Difficulties of emotion regulation scale; MAIA, Multidimensional assessment of interoceptive awareness; ITC-SOPI, Experience in the use of technologies. A brief version of the Independent Television Company SOP Inventory; SPQ, Sense of presence; GSR, galvanic skin response; EEG, electroencephalogram; MPQ, The McGill Pain Questionnaire; PASS, Pain anxiety symptoms scale; PCS, Pain catastrophizing scale; PSEQ, Pain self-efficacy questionnaire; PACU, post-anesthesia recovery unit; SNDD, the SD of normal to norma; VASA, invasive coronary angiography; ICA, invasive coronary angiography

The most commonly measured outcomes are related to psychological or physiological indicators. Psychological indicators are primarily assessed through questionnaire-based ratings obtained via patient self-reports or observations by healthcare professionals such as doctors or nurses. The measured outcomes mainly include pain, stress, anxiety, satisfaction, and depression. Physiological indicators are primarily measured using various instruments to assess cardiovascular or endocrine parameters (as signs of stress or relaxation). The measurement tools for outcomes can serve as effective indicators of the impact of nature in virtual reality on patients.

**Fig 4:**
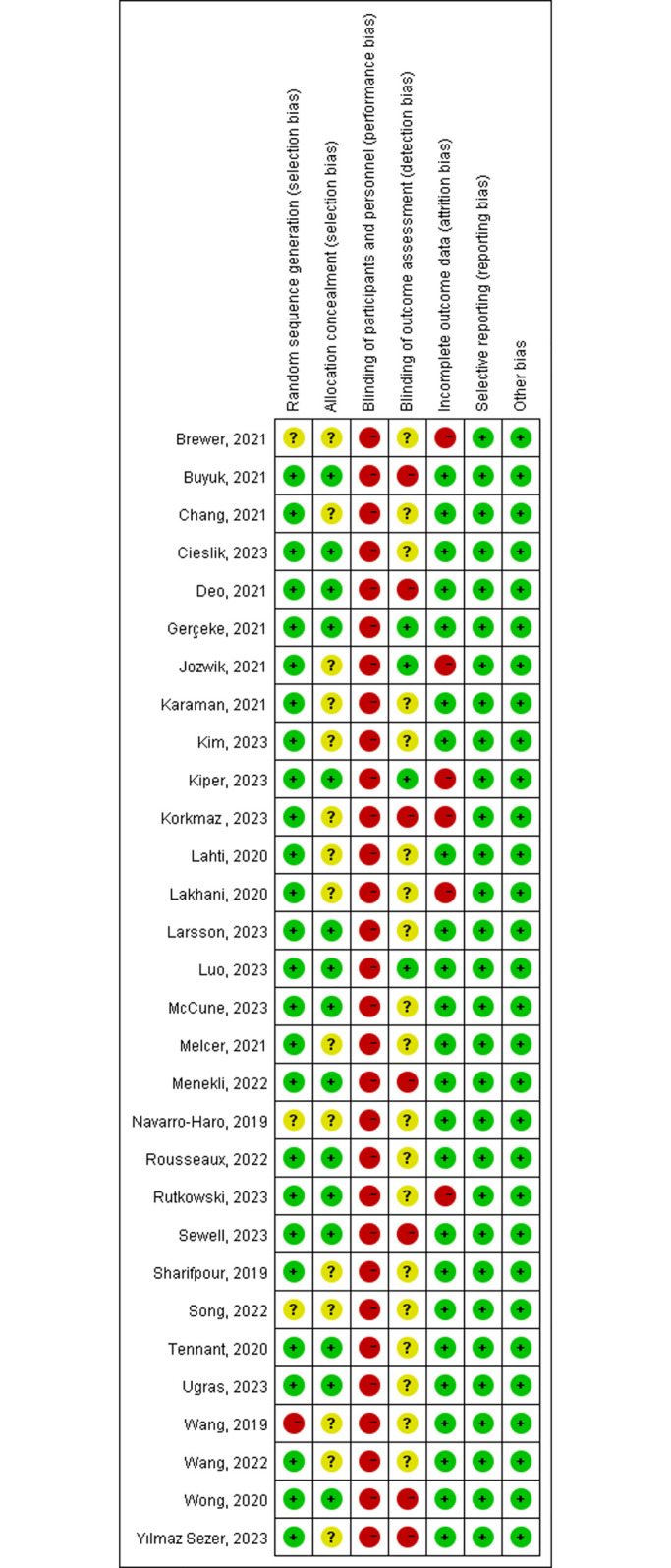
Risk of bias summary: authors’ judgments about each item of the risk of bias assessment for every included study. “+” indicates low risk of bias; “-” indicates high risk of bias; “?” indicates unclear or unknown risk of bias.

**Fig 5:**
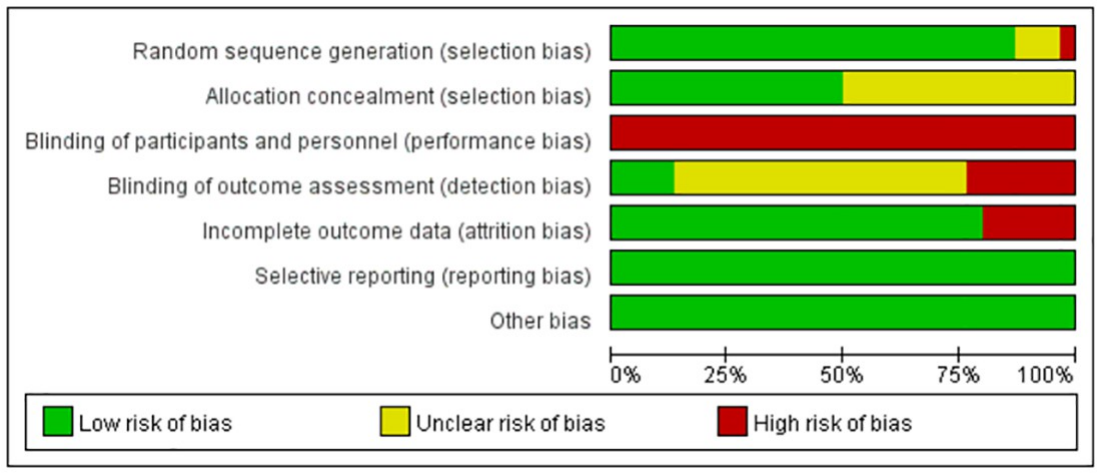
Risk of bias graph: authors’ judgments about each item of the risk of bias assessment presented as percentages among all included studies.

Fig 4 summarizes the bias assessment results for all studies. The majority of results clearly reported the method of random sequence generation. Three studies only reported random allocation of patients without detailing the randomization process. One study allocated participants based on gender and physical activity levels, posing a high risk of bias. Fifteen studies specified allocation concealment. Due to the nature of the interventions, blinding of participants and researchers was not possible. However, four studies explicitly stated that outcome assessments were conducted in a blinded manner. Most included trials provided complete data or missing data did not affect the pooled meta-analysis results, with only six trials providing partial data due to imbalanced subject withdrawals, among other reasons. The risks of selective reporting and other biases were low. Overall (Fig 5), the risks of bias for Random Sequence Generation, Incomplete Outcome Data, Selective Reporting, and Other Bias were low. The risks of Allocation Bias, Concealment of Outcome Assessment, and Blinding of Participants and Personnel remain unclear. The risk of bias due to blinding of participants and personnel was high. Across all included studies, the overall risk of bias ranged from low to moderate.

### 4 Main outcome and meta-analysis of outcome measure

#### 4.1 Effects of virtual nature on pain

**Fig 6:**
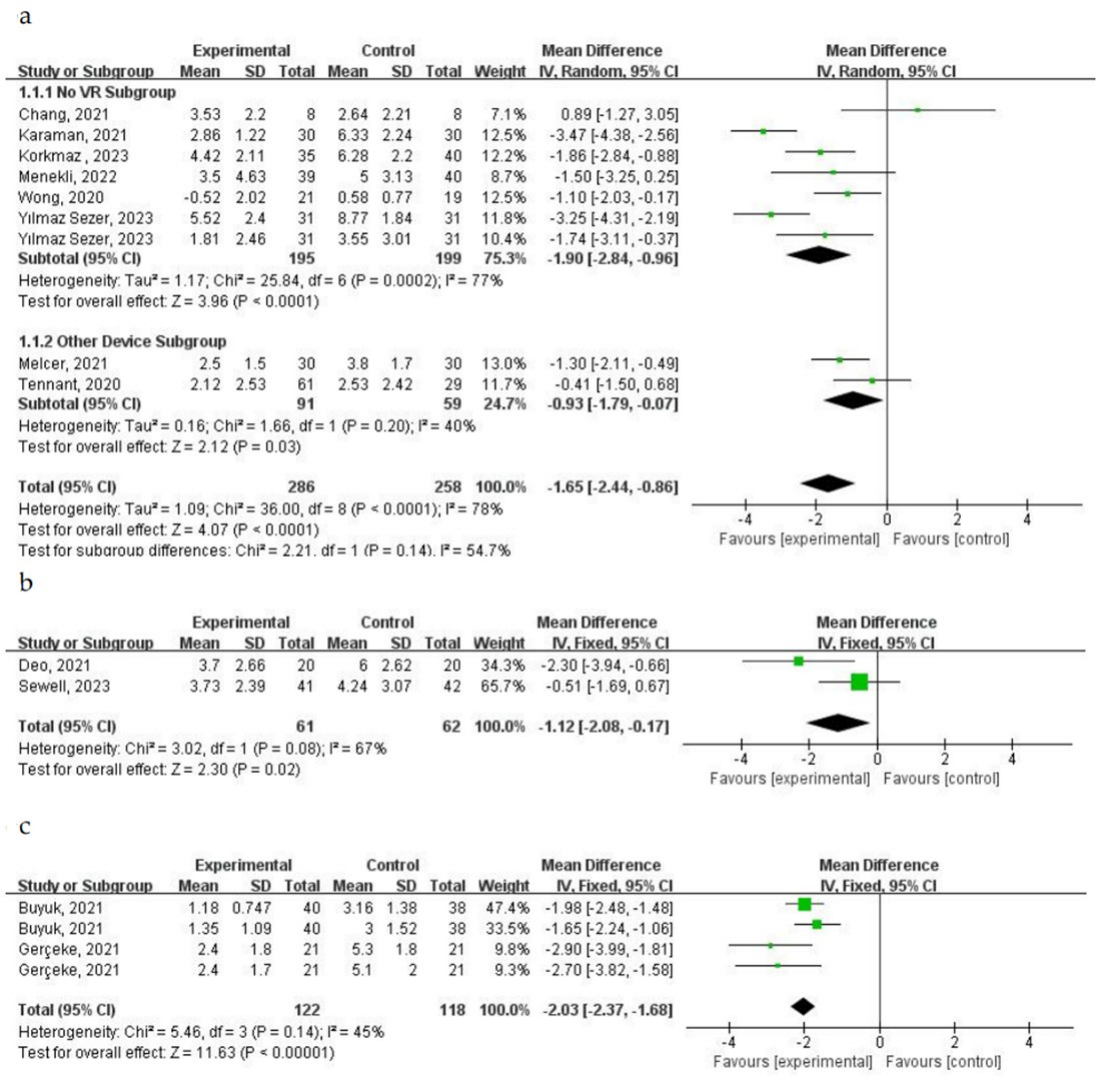
Forest plot for meta-analysis of primary outcomes related to pain. Mean difference (95% CI) of the effect of VR-based rehabilitation (experimental group) compared with conventional treatment (control group) on a VAS, b NRS, and c WBS. CI confidence interval, SD standard deviation.

A total of 20 studies assessed the impact of immersion in virtual nature on patients’ pain perception. Among these, 8 studies involving 392 patients reported pain intensity using the Visual Analog Scale (VAS) (Fig 6a). Three studies (McCune, 2023; Rousseaux, 2022; Kiper, 2023) were not included in the analysis due to unextractable data. The studies were categorized into subgroups: No VR Subgroup and Other Device Subgroup (using other devices to view the same natural scenes as in VR) in the control group. Heterogeneity analysis revealed the following results: P < 0.0001 and I^2^ = 78% for all 8 studies, P = 0.0002 and I^2^ = 77% for the No VR Subgroup, P = 0.2 and I^2^ = 40% for the Other Device Subgroup. A random-effect model was selected due to I^2^ > 50%. The pooled mean difference was -1.65 (-2.44, -0.86), p < 0.0001. The results suggest a statistically significant overall reduction in pain intensity scores during VR-assisted therapy compared to conventional treatment or using other devices. Statistically significant differences were also observed in both the No VR Subgroup and the Other Device Subgroup (MD = -1.9 (-2.84, -0.96) and P < 0.0001, MD = -0.93 (-1.79, -0.07) and P = 0.03, respectively). The remaining three studies using VAS, which were not included in the meta-analysis, involved 190 patients, and no statistically significant differences in pain scores were observed at any time point (p > 0.05).

Two studies involving 123 patients reported pain intensity using the Numeric Rating Scale (NRS) (Fig 6b). The NRS scores in the VR-based rehabilitation group were significantly lower compared to the control group (MD = -1.12 (-2.08, -0.17) and P = 0.02). There was high heterogeneity (I^2^ = 67%, P = 0.08), thus a random-effect model was chosen as I^2^ > 50%.

Four studies involving 120 patients reported pain intensity using the Wong-Baker FACES (WBS) Pain Rating Scale (Fig 6c). One study (Brewer, 2021) was not included in the analysis due to unextractable data. The WBS scores in the VR-based rehabilitation group were significantly lower compared to the control group (MD = -2.03 (-2.37, -1.68) and P < 0.01). There was moderate heterogeneity (I^2^ = 45%, P = 0.14), thus a fixed-effect model was chosen as I^2^ < 50%. Brewer (2021) reported no statistically significant difference in pain scores between the control and experimental groups among the 40 patients.

Two additional studies employed different assessment scales to gauge pain intensity. In the study conducted by Luo (2023), pain intensity was assessed in a sample of 106 patients divided into the Biophilic Virtual Reality Group (BVG), Indoor Virtual Reality Group (IVG), and Blank Control Group (BCG). The assessment utilized the Revised Faces Pain Scale (FPS-R) and Pain Index (Pi). Results indicated that the FPS-R scores in both the BVG and IVG were significantly lower than those in the Blank Control Group (P < 0.05). However, there were no significant differences in FPS-R scores between the BVG and IVG (P = 1.000). Regarding the Pi scores, there were no notable disparities between the BVG and IVG at any point during surgery and the postoperative period (T3-T6), with the BVG Pi scores only registering lower than those in the Blank Control Group at T5 (P < 0.05). Furthermore, the study unveiled a significant correlation between Pi scores and VAS scores.

In the research conducted by Sharifpour (2019), pain intensity, pain anxiety, pain catastrophizing, and pain self-efficacy were assessed in a cohort of 30 adolescents. This evaluation employed the McGill Pain Questionnaire (MPQ), Pain Anxiety Symptoms Scale (PASS), Pain Catastrophizing Scale (PCS), and Pain Self-Efficacy Questionnaire (PSEQ). Notably, significant differences in pain-related variables were observed between the control and experimental groups at the post-test, first follow-up, and second follow-up stages (p < 0.01).

#### 4.2 Effects of virtual nature on anxiety

**Fig 7:**
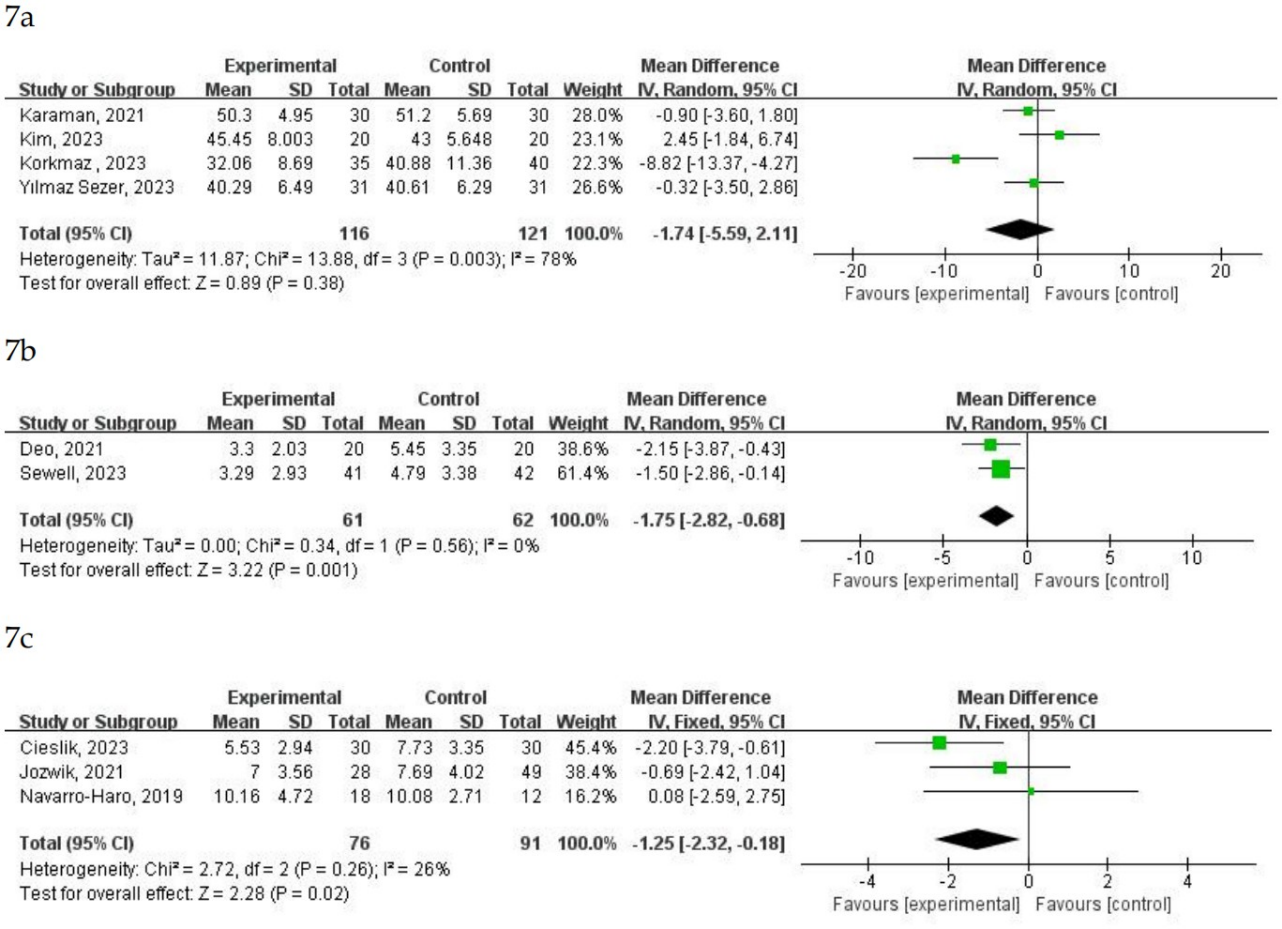
Forest plot for meta-analysis of primary outcomes related to anxiety. Mean difference (95% CI) of the effect of VR-based rehabilitation (experimental group) compared with conventional treatment (control group) on a STAI, b HANDS, and c NRS. CI confidence interval, SD standard deviation.

A total of 23 studies assessed the impact of immersion in virtual nature on patients’ anxiety levels. Four studies involving 237 patients reported anxiety levels using the State and Trait Anxiety Inventory (STAI) (Fig 7a). Two studies (Menekli, 2022; Melcer, 2021) were not included in the analysis due to unextractable data. The results indicated no statistically significant difference between the experimental and control groups (MD = -1.74 (-5.59, 2.11) and P = 0.38). However, there was high heterogeneity (I^2^ = 78%, P = 0.003). A random-effect model was chosen as I^2^ > 50%. Melcer (2021) categorized preoperative and postoperative anxiety levels as low, moderate, and high. The STAI scores of 60 patients in both the control and experimental groups showed no significant differences (P > 0.05). Menekli (2022) reported a significant decrease in STAI scores for the experimental group compared to the control group among 139 patients (p < 0.001).

Two studies involving 123 patients reported anxiety levels using NRS (Fig 7b). The NRS scores in the VR-based rehabilitation group were significantly lower compared to the control group (MD = -1.75 (-2.82, -0.68) and P = 0.001). No heterogeneity was found (I^2^ = 0%, P = 0.56). A fixed-effect model was chosen as I^2^ < 50%.

Three studies involving 167 patients reported anxiety levels using the Hospital Anxiety and Depression Scale Anxiety (HADS) (Fig 7c). One study (Kiper, 2023) was not included in the analysis due to unextractable data. The HADS scores in the VR-based rehabilitation group were significantly lower compared to the control group (MD = -1.25 (-2.32, -0.18) and P = 0.02). The heterogeneity in the included studies was not significant (I^2^ = 26%, P = 0.26). A fixed-effect model was chosen as I^2^ < 50%. Kiper (2023) reported that among 60 patients, the experimental group’s HADS-A scores were significantly lower than those in the control group (p < 0.01).

Additionally, 14 studies not included in the meta-analysis assessed the level of anxiety. Luo (2023) reported anxiety levels using the simplified Chinese version of the modified Yale Preoperative Anxiety Scale (CmYPAS), Visual Analogue Scale (VAS), Heart rate (HR), and Anxiety index (Ai) for the biophilic virtual reality group (BVG), indoor virtual reality group (IVG), and blank control group (BCG). The CmYPAS scores, VAS scores, and Ai scores of the BVR group were significantly lower than those of the blank control group (P<0.01), while there was no statistical difference in HR scores between the BVR group and IVR group (P>0.05). There were no differences in CmYPAS scores, VAS scores, and Ai scores between the BVR group and IVR group (P>0.05). Ugras (2023) reported anxiety levels using the Anxiety Specific to Surgery Questionnaire (ASSQ), showing significant differences in ASSQ scores between the experimental and control groups at different time points (P<0.001). Kiper (2023) reported anxiety levels using HADS, indicating significantly lower HADS-A scores in the experimental group compared to the control group (p<0.01). Menekli (2022) reported anxiety levels using VAS, showing significantly lower VAS scores in the experimental group compared to the control group (p<0.001). Rousseaux (2022) reported anxiety levels using VAS, indicating significant differences between the VR group and hypnosis group in baseline differences and duration (P < 0.05), while there were no significant differences between the two groups at any other time (P>0.05). Melcer (2021) reported anxiety levels using STAI scores and classified anxiety populations by Anxiety level, showing no significant differences in STAI scores between the control group and experimental group (P>0.05). Chang (2021) reported anxiety levels using Subjective Units of Distress (SUDS), indicating significantly lower SUDS scores in the experimental group compared to the control group (p<0.01). Buyuk (2021) reported children’s anxiety levels using the children’s anxiety meter scale (CAM-S), showing significantly lower CAM-S scores in the experimental group compared to the control group (p<0.001). Gerçeke (2021) reported anxiety levels using CAS-D, showing significantly lower CAS-D scores in the experimental group compared to the control group (p<0.001). Brewer (2021) reported anxiety levels using the visual facial anxiety scale, showing significantly lower VFAS scores in the experimental group compared to the control group (p<0.001). Tennant (2020) reported anxiety levels using VAS scores for VR use and viewing natural pictures on an iPad, showing no significant differences in VAS scores between the control group and experimental group (P>0.05). Lahti (2020) utilized the Modified Dental Anxiety Scale (MDAS) to assess the level of anxiety, where the decline in MDAS scores was statistically significant only among females in the VRR group. Larsson (2023) reported anxiety levels using the Visual Analog Scale (VAS), indicating that before vascular puncture, immediately following the use of VR headphones, the experimental group’s VAS scores were significantly lower than those of the control group (p<0.05).

#### 4.3 Effects of virtual nature on depression

**Fig 8:**
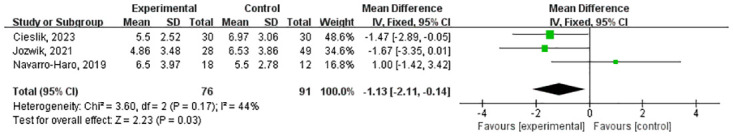
Forest plot for meta-analysis of primary outcomes related to depression. Mean difference (95% CI) of the effect of VR-based rehabilitation (experimental group) compared with conventional treatment (control group) on HANDS. CI confidence interval, SD standard deviation.

A total of 6 studies evaluated the impact of immersion in virtual nature on patients’ depression. Three studies involving 167 patients reported depression levels using the Hospital Anxiety and Depression Scale (HADS) (Fig 8). The HADS scores in the VR-based rehabilitation group were significantly lower compared to the control group (MD = -1.13 (-2.11, -0.14) and P = 0.03). There was moderate heterogeneity (I^2^ = 44%, P = 0.17). A fixed effect model was chosen as I^2^ < 50%.

Additionally, three studies also assessed depression levels. Cieslik (2023) and Kiper (2023) reported depression levels in 120 patients using The 30-item Geriatric Depression Scale (GDS-30), and the results showed significantly lower GDS scores in the control group compared to the experimental group (p < 0.01). Song (2022) reported patients’ stress using the Self-Rating Depression Scale (SDS), and the results indicated no significant differences in PSS scores between the control and experimental groups (P > 0.05).

#### 4.4 Effects of virtual nature on fear

**Fig 9:**
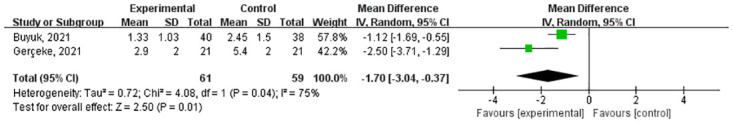
Forest plot for meta-analysis of primary outcomes related to fear. Mean difference (95% CI) of the effect of VR-based rehabilitation (experimental group) compared with conventional treatment (control group) on CFS. CI confidence interval, SD standard deviation.

Two studies involving 120 patients reported fear levels using the Children’s Fear Scale (CFS) (Fig 9). The CFS scores in the VR-based rehabilitation group were significantly lower compared to the control group (MD = -1.70 (-3.04, -0.37) and P = 0.01). However, there was high heterogeneity (I^2^ = 75%, P = 0.04). A random effect model was chosen as I^2^ > 50%.

Additionally, one study not included in the meta-analysis assessed fear levels. Yılmaz Sezer (2023) reported fear levels using VAS, and the average fear scores showed no significant difference between the experimental and control groups (p = 0.362). However, the most intense fear in the experimental group was significantly lower than in the control group (P < 0.05).

#### 4.5 Effects of virtual nature on satisfaction

A total of 6 studies evaluated patient satisfaction with VR. Sewell (2023), Chang (2023), Yılmaz Sezer (2023), Larsson (2023), and McCune (2023) reported patient satisfaction using various scales in a total of 348 patients. The results indicated no significant differences in satisfaction scores between the control and experimental groups (P > 0.05), but the satisfaction levels in the VR group were generally higher than in the control group. Kim (2023) reported patient satisfaction using VAS in a group of 40 patients, and the results showed significantly lower VAS scores in the experimental group compared to the control group (p < 0.05).

#### 4.6 Effects of virtual nature on stress

A total of 3 studies assessed patients’ stress levels. Rutkowski (2023) and Song (2022) reported stress levels in a total of 95 patients using The Perceived Stress Scale (PSS-10) and The Self-Rating Depression Scale (SDS), respectively. The results showed no significant differences in stress scores between the control and experimental groups (P > 0.05). Jozwik (2021) reported stress levels in 77 patients using The Perception of Stress Questionnaire (PSQ), and the results indicated significantly lower PSQ scores in the experimental group compared to the control group (p < 0.001).

#### 4.7 Effects of virtual nature on Diastolic Blood Pressure (DBP)

**Fig 10:**
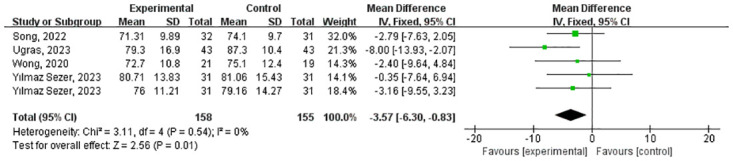
Forest plot for meta-analysis of primary outcomes related to DBP. Mean difference (95% CI) of the effect of VR-based rehabilitation (experimental group) compared with conventional treatment (control group). CI confidence interval, SD standard deviation.

Five studies involving 251 patients reported DBP (Fig 10). DBP in the VR-based rehabilitation group was significantly lower compared to the control group (MD = -3.57 (-6.30, -0.83) and P = 0.01). No heterogeneity was observed (I^2^ = 0%, P = 0.54). A fixed effect model was chosen as I^2^ < 50%.

Menekli (2022) was excluded from the meta-analysis due to higher heterogeneity compared to other experiments. This study involved 139 individuals and measured preoperative, intraoperative, and postoperative DBP. While there was no significant difference in DBP between the experimental and control groups preoperatively (p = 0.068), the experimental group exhibited significantly lower DBP during (p<0.001) and after the surgery (p<0.001) compared to the control group.

#### 4.8 Effects of virtual nature on Systolic Blood Pressure (SBP)

**Fig 11:**
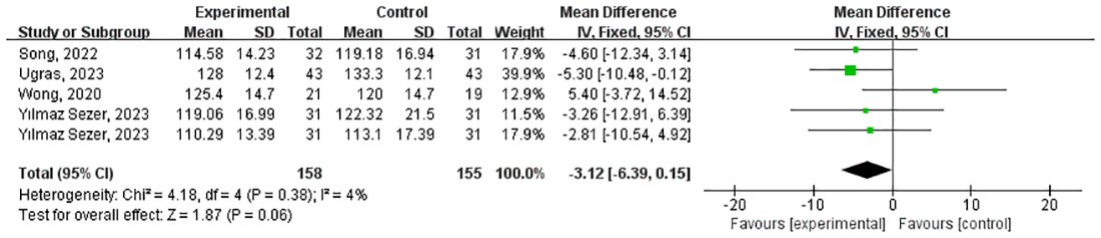
Forest plot for meta-analysis of primary outcomes related to SBP. Mean difference (95% CI) of the effect of VR-based rehabilitation (experimental group) compared with conventional treatment (control group). CI confidence interval, SD standard deviation.

Five studies involving 251 patients reported SBP (Fig 11). There was no statistically significant difference in SBP between the experimental and control groups (MD = -3.12 (-6.39, 0.15) and P < 0.0001). The heterogeneity in the included studies was not significant (I^2^ = 4%, P = 0.38). A fixed effect model was chosen as I^2^ < 50%.

Menekli (2022) was excluded from the meta-analysis due to higher heterogeneity compared to other experiments. This study involved 139 individuals and measured preoperative, intraoperative, and postoperative SBP. While there was no significant difference in SBP between the experimental and control groups preoperatively (p = 0.251), the experimental group exhibited significantly lower SBP during (p<0.001) and after the surgery (p<0.001) compared to the control group.

#### 4.9 Effects of virtual nature on Heart Rate (HR)

**Fig 12:**
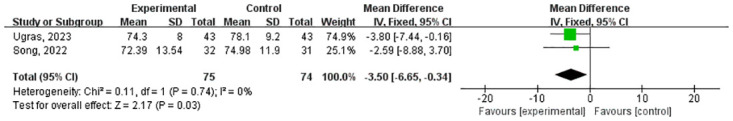
Forest plot for meta-analysis of primary outcomes related to HR. Mean difference (95% CI) of the effect of VR-based rehabilitation (experimental group) compared with conventional treatment (control group). CI confidence interval, SD standard deviation.

Three studies involving 288 patients reported HR (Fig 12). HR in the VR-based rehabilitation group was significantly lower compared to the control group (MD = -3.50 (-6.65, -0.34) and P = 0.03). No heterogeneity was observed (I^2^ = 0%, P = 0.74). A fixed effect model was chosen as I^2^ < 50%.

Menekli (2022) was excluded from the meta-analysis due to higher heterogeneity compared to other experiments. This study involved 139 individuals and measured preoperative, intraoperative, and postoperative HR. While there was no significant difference in HR between the experimental and control groups preoperatively (p = 0.3), the experimental group exhibited significantly lower HR during (p<0.001) and after the surgery (p<0.001) compared to the control group.

#### 4.10 Effects of virtual nature on SpO_2_

**Fig 13:**
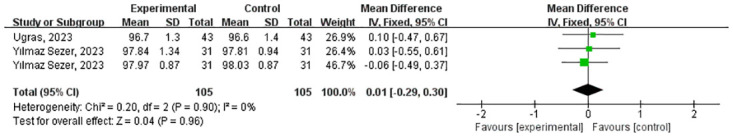
Forest plot for meta-analysis of primary outcomes related to SpO2. Mean difference (95% CI) of the effect of VR-based rehabilitation (experimental group) compared with conventional treatment (control group). CI confidence interval, SD standard deviation.

Three studies involving 148 patients reported SpO_2_ (Fig 13). There was no statistically significant difference in SpO_2_ between the experimental and control groups (MD = -0.01 (-0.29, 0.30) and P = 0.96). No heterogeneity was observed (I^2^ = 0%, P = 0.90). A fixed effect model was chosen as I^2^ < 50%.

Menekli’s (2022) study was not included in the meta-analysis due to higher heterogeneity compared to other studies. This experiment involved 139 individuals and measured SpO2 levels preoperatively, intraoperatively, and postoperatively. The SpO2 levels between the experimental and control groups showed no significant differences preoperatively (p = 0.363) and postoperatively (p = 0.153). However, during the surgery, the experimental group exhibited significantly lower SpO2 levels (p<0.001) compared to the control group.

### 5. Effects of virtual natural scenes on patients

Table 4 demonstrates the effectiveness of various virtual natural scenes included in the articles on multiple patient outcome measures. There is only one study using desert scenes, comprising a sample size of 60 individuals. This study indicated that the desert natural environment effectively alleviated pain but did not reduce anxiety. Two articles measuring the impact of climate scenes, with a combined sample size of 130 individuals, showed contrasting results in alleviating pain and anxiety, with more results leaning towards no significant impact. There are five articles using Mountain scenes, totaling 370 individuals, demonstrating contrasting outcomes in alleviating pain and anxiety, with more results indicating no significant effects. Nine articles employing Forest scenes (excluding Wang (2022) as it did not compare VR with non-VR interventions and hence was not included in the table), totaling 597 individuals, showed effectiveness in alleviating pain, anxiety, and fear while enhancing satisfaction. However, contradictory outcomes were observed in physiological indicators, with more results leaning towards no significant impact. There are 22 articles using Water scenes (excluding Wang, 2022), totaling 1501 individuals, demonstrating efficacy in alleviating pain, anxiety, and fear while enhancing satisfaction. However, contradictory outcomes were noted in physiological indicators. Twenty-two articles employing Gardens scenes (excluding Wang, 2022), totaling 950 individuals, showed effectiveness in alleviating pain, anxiety, and depression while enhancing satisfaction and effectively improving physiological indicators.

**Table 4:** The therapeutic effects of different intervention scenarios.

Study	Sample size	Reduce Pain	Reduce Anxiety	Reduce Depression	Reduce Fear	Enhance Satisfaction	Reduce Stress	DBP	SBP	HR	SpO_2_
Climate											
Rousseaux, 2022	100	-	-							-	
Sharifpour, 2019	30	+	+								
Total	130										
Desert											
Melcer, 2021	60	+	-								
Total	60										
Mountain											
Larsson, 2023	156		-			-					
McCune, 2023	30	-				-					
Melcer, 2021	60	+	-								
Lakhani, 2020	24	+	+								
Rousseaux, 2022	100	-	-							-	
Total	370										
Forest											
Larsson, 2023	156		-								
McCune, 2023	30	-				-					
Wong, 2020	40	+						-	-	-	
Deo, 2021	40	+	+								
Gerçeke, 2021	42	+	+		+						
Buyuk, 2021	78	+	+		+						
Song, 2022	63							-	-	-	
Ugras, 2023	86		+					+	+	+	-
Yılmaz Sezer, 2023	62	+	-		+	-		-	-		-
Total	597										
Water scenes											
Larsson, 2023	156		-			-					
McCune, 2023	30	-				-					
Sharifpour, 2019	30	+	+								
Navarro-Haro, 2019	39		-	-							
Lakhani, 2020	24	+	+								
Wong, 2020	40	+						-	-	-	
Lahti, 2020	255		+								
Deo, 2021	40	+	+								
Brewer, 2021	40		+								
Gerçeke, 2021	42	+	+		+						
Buyuk, 2021	78	+	+		+						
Karaman, 2021	60	+	-								
Chang, 2021	15	-	+			-					
Rousseaux, 2022	100	-	-							-	
Melcer, 2021	60	+	-								
Menekli, 2022	139	-	+					+	+	+	+
Song, 2022	63							-	-	-	
Ugras, 2023	86		+					+	+	+	-
Yılmaz Sezer, 2023	62	+	-		+	-		-	-		-
Korkmaz, 2023	70	+	+								
Kim, 2023	40	-	-			+					
Rutkowski, 2023	32						-				
Total	1501										
Gardens											
Wang, 2019	60			+						-	
Lakhani, 2020	24	+	+								
Tennant, 2020	90	-	-								
Jozwik, 2021	77		-	-			+				
Menekli, 2022	139	-	+					+	+	+	+
Song, 2022	63							-	-	-	
Kiper, 2023	60	-	+	+							
Ugras, 2023	86		+					+	+	+	+
Korkmaz, 2023	70	+	+								
Sewell, 2023	83	-	+			-					
Luo, 2023	106	+	+								
Cieslik, 2023	60		+	+							
Rutkowski, 2023	32						-				
Total	950										

"+" signifies a significant impact of the intervention involving virtual natural scenes on a specific measurement aspect within the article. "-" denotes no significant impact of the intervention involving virtual natural scenes on a specific measurement aspect within the article.

Five articles compared the impact of immersing patients in virtual natural environments in the experimental group with immersing them in virtual gray spaces in the control group on patient health. In Song’s (2022) study, a comparison was made between blue spaces, green spaces with different levels of openness, and the indoor gray space of a hospital. The results showed that cancer patients had the highest preference for blue space, followed by open green space, closed green space, semi-open green space, and finally, the hospital building environment’s gray space. Wang (2019) compared cycling while simultaneously viewing a virtual park road with cycling while simultaneously viewing virtual watercolor paintings. The results showed that the experimental group had higher exercise intensity and lower emotional stress. In his 2022 experiment, Wang compared the impact of different tree density on visual behavior in adults with generalized anxiety disorder. The results showed that exercising under moderate tree density best reduced stress, while exercising under higher tree density resulted in more virtual attention. However, two other studies provided different conclusions. Both Luo (2023) and Rousseaux (2022) showed that there was no significant difference in the impact on patients’ health between viewing natural landscapes and indoor landscapes in VR.

## Discussion

This study aims to complement traditional medical approaches and enhance environmental design in the field of public health, conducted through a systematic review and meta-analysis of randomized controlled trials. A total of 30 articles were included in this study, primarily assessed using psychological and physiological indicators. Among them, 27 articles each demonstrated that immersion in virtual natural environments significantly affects patient recovery in the context of landscape design management. Natural scenes including blue and green elements have been applied more extensively and have shown more significant effects. That demonstrates their potential to reshape medical interventions and improve environmental design in the field of public health.

The various analyses conducted in this study underscore the pivotal role of virtual reality in landscape design management. Through VR technology, patients can immerse themselves in captivating natural environments, thereby alleviating pain, anxiety, depression, and fear, among other discomforts. The visual and auditory effects of VR enable patients to shift their focus to different sensory domains, thus mitigating negative emotions and anxiety, enhancing positive emotions, and providing a sense of satisfaction and relaxation [37]. This aligns with the meta-analysis results in this paper, where by redirecting patients’ attention, VR leads to higher pain thresholds, increased pain tolerance, and reduced pain intensity [54, 55]. Pain and anxiety are closely related [54], and individuals with higher levels of anxiety may experience higher degrees of pain during invasive procedures, as they share biological pathways and neurotransmitters [56].

However, there are also exceptions. Rousseaux (2022) indicates that the pain and anxiety relief effects were similar in the control group, hypnotherapy group, VR intervention group, and VR and hypnotherapy group. This suggests that traditional drug therapy may already be sufficiently effective for these patients, possibly because the researchers visited the patients before surgery to reassure them and reduce anxiety, regardless of the technology used. Tennant (2020) found that the effects of virtual reality and iPad intervention were similar in relieving pain, alleviating anxiety, and increasing happiness. This may be because patients reported similar subjective immersion levels in both cases, but higher levels of VR immersion consistently favored VR in all subjective measurements.

VR’s effectiveness in alleviating patient stress and enhancing patient satisfaction remains inconclusive at present due to a lack of support from existing data for meta-analyses. Both outcomes have been reported in studies included, but there’s a scarcity of articles and sample sizes. There are only three articles measuring stress levels: Jó zwik (2021) and Rutkowski (2023) designed experiments with VR interventions compared to standard rehabilitation training, while Song (2022) focused on natural scenes versus indoor spaces within VR. The efficacy of rehabilitation within VR seems comparable to traditional training, yet the differing results in Jó zwik (2021) and Rutkowski (2023) might stem from variances in rehabilitation training or the effects of VR experiences. These findings align somewhat with Suseno (2023), suggesting no discernible therapeutic differences between 2D videos and virtual reality, potentially due to environmental features, display devices, and other factors [57]. Furthermore, Song’s (2022) research indicates a certain stress alleviation for patients viewing grey spaces within VR, possibly suggesting gradual relaxation for patients in everyday life without any interventions. However, natural environments might enhance psychological and emotional recovery capabilities. Regarding patient satisfaction, the VR groups in the majority of studies reported significantly higher satisfaction with surgery compared to the control groups [37, 38]. Evidence supporting the effectiveness of Virtual Forest Therapy (VFT) in stress relief was provided in Abdullah et al.’s systematic review in 2017. While patient satisfaction with surgery or examinations did not significantly differ from the control group in some studies [27], there was a high rate of recommendation and favorability. This indicates that further research is needed to confirm these findings.

One notable observation is the diversity in the types of virtual natural scenes utilized in the interventions. The categorization of scenes allows for a comprehensive exploration of their impact on patients and provides guidance for future environmental designs. Interestingly, there is a parallel trend between the quantity of applications featuring natural scenes and their effectiveness in patient healing. Desert and mountain landscapes are among the least applied and show inconsistent therapeutic effects. Conversely, water scenes, forest landscapes, and garden views are more frequently applied and exhibit more notable effects. This might be correlated with landscape preferences. The prevalence of aquatic landscapes, including islands, seascapes, beaches, and waterfalls, suggests a preference for serene and calming environments. This aligns with previous research highlighting the therapeutic benefits of water-based scenes in reducing stress and anxiety [58]. The inclusion of garden or park landscapes in 14 instances indicates a recognition of the soothing effects of natural green spaces. This choice may resonate with patients seeking a more familiar and comforting environment. Similarly, the use of natural forest or woodland landscapes in eleven studies acknowledges the well-documented benefits of nature exposure on mental well-being and stress reduction [1]. The studies involving five mountainous landscapes and one desert landscape may reflect an interest in providing patients with awe-inspiring and visually stimulating experiences. Mountains are renowned for their ability to evoke grandeur and wonder, while the interest in desert landscapes might signify a psychological fascination with exploring barren and open environments, potentially enhancing the immersive nature of VR interventions [59]. This immersion can effectively distract and alleviate pain but might not necessarily relax patients’ anxious minds. However, current research regarding the therapeutic potential of such landscape applications remains relatively limited and requires further investigation.

In 5 studies comparing virtual natural environments with virtual gray spaces, 3 studies suggested that immersion in virtual nature was more effective for patient recovery, with a higher preference for blue spaces and open green spaces. The "prospect-refuge theory" proposes that picturesque bodies of water and neatly manicured lawns in open landscapes provide no hiding places for predators, offering an increased sense of safety [60]. This aligns with instinctive judgments of early humans [61]. Other studies using VR greenery have also shown similar effectiveness [62]. Different VR environments based on nature are useful for improving mood [63]. Exercising in indoor virtual environments can help patients establish and maintain regular exercise habits, without being deterred by rain, severe air pollution, or a lack of natural scenery [40]. Studies showing no significant differences should be noted, and future research with more precise methodologies and larger sample sizes will be needed to assess the impact of natural vs. gray spaces in VR.

Our review possesses numerous strengths that enhance the credibility and depth of our findings. Firstly, all 30 studies adhered to a randomized controlled trial design, demonstrating a spectrum of bias risks ranging from low to moderate. This design choice bolsters the methodological rigor of our review. Secondly, the incorporation of a substantial number of studies and participants augments the statistical robustness of our analyses, lending greater persuasiveness to our research outcomes. Furthermore, these studies encompassed various pain states and employed diverse psychological measurement tools, such as VAS, NRS, and HADS, thus enriching our dataset and facilitating a more comprehensive grasp of the impact of virtual nature on patients across multiple dimensions. Lastly, we conducted a meticulous and systematic synthesis and analysis of a substantial body of research encompassing a wide array of assessments related to pain, anxiety, depression, and fear. This thorough approach further elevates the study’s credibility.

Nonetheless, our review is not without limitations. In certain analyses, we observed substantial or moderate heterogeneity, indicating differences among the studies. These variations may be attributed to factors such as research methodologies and sample characteristics, necessitating a nuanced interpretation of the results. Moreover, the variability in measurement tools employed across some studies precluded their inclusion in the meta-analysis, potentially influencing the outcomes related to patients’ immersion in virtual nature. It’s important to note that some studies omitted measurement time in their analysis, which could lead to inconsistencies when comparing findings across different studies. Despite our inclusion of a substantial number of studies, there is a possibility that unpublished or grey literature studies were overlooked, introducing the potential for publication bias.

Another limitation to acknowledge is that the majority of studies reviewed here primarily investigated the short-term effects of virtual nature, with relatively fewer examining its long-term impacts. Consequently, our understanding of the enduring effects of virtual nature on patients may be somewhat constrained. Furthermore, our paper did not delve into the influence of individual differences, such as age, gender, and cultural background, on the effects of virtual nature, factors which could potentially exert a substantial impact on research outcomes.

In forthcoming research endeavors, Virtual Reality (VR) technology emerges as a pivotal factor in the realm of public health and environmental design. Firstly, there exists significant potential in delving deeper into the study of how various types of virtual natural environments impact patients, aiming to discern the most effective therapeutic outcomes. This involves investigating the application effects of specific natural settings, such as forests, aquatic environments, mountains, etc., across diverse therapeutic contexts and understanding their distinct influences on the psychological and physiological states of patients. Secondly, the ongoing evolution of technology allows for the optimization of VR hardware and software, ushering in more realistic, immersive, and personalized experiences [64]. This optimization may involve incorporating advanced sensor technologies, refining audio-visual effects, and enhancing interaction methods to amplify the sense of engagement and enjoyment within the virtual environment. Moreover, future research can center on exploring the synergistic application of VR with other therapeutic approaches, including music therapy, art therapy, etc., to further augment therapeutic effects. By integrating diverse therapeutic methods, more comprehensive and personalized treatment plans can be devised to cater to the specific needs of varied patient groups. Finally, it is imperative to address sustainability and practical application concerns associated with VR in therapeutic design and management. This encompasses considerations regarding equipment costs, maintenance, updates, and the integration of VR technology into clinical practice.

## Conclusions

In conclusion, this research of 30 randomized controlled trials substantiates the profound impact of immersing individuals in virtual natural environments within the realm of therapeutic landscape design management. The results demonstrate significant reductions in pain intensity and anxiety levels, highlighting the potential of VR therapy in alleviating negative emotional states. Furthermore, patients exposed to virtual natural scenes reported lower levels of depression and fear, underscoring the therapeutic benefits of integrating virtual nature into landscapes. While no statistically significant differences were observed in SpO_2_ and patient satisfaction, this calls for further exploration in these domains.

It is important to acknowledge the diversity in methodologies, sample characteristics, and measurement tools across the studies, contributing to some level of heterogeneity in the findings. Additionally, the majority of studies primarily focused on short-term effects, warranting more research on the enduring impacts of virtual nature on individuals. Individual differences, including age, gender, and cultural background, may also play a significant role in shaping the effects of virtual nature, an aspect deserving of further investigation.

In light of these considerations, the incorporation of virtual nature in landscape design management emerges as a promising approach for enhancing well-being and quality of life. Future research endeavors should address the identified gaps and provide a more nuanced understanding of the specific contexts and populations that may benefit most from this intervention. This investigation firmly advocates for VR as a pivotal tool in augmenting the therapeutic potential of landscapes. It highlights how VR’s immersive capabilities have the potential to revolutionize the field of therapeutic landscape design and management, offering innovative and effective interventions for enhancing human well-being. As this field progresses, further research should focus on long-term follow-ups and standardized protocols to unlock the full spectrum of benefits this innovative intervention promises.

## Supporting information

S1 Checklist PRISMA 2020 flow diagram. (DOCX)

S2 Checklist PRISMA 2020 checklist. (DOCX)
