# Supplementary material for: Improving immersive experiences in virtual natural setting for public health and environmental design: A systematic review and meta-analysis of randomized controlled trials
Source: PLoS One. 2024 Apr 17;19(4):e0297986. doi: 10.1371/journal.pone.0297986 (PMC11023440; doi:10.1371/journal.pone.0297986)
Supplement: S1 Checklist — (DOCX) [file pone.0297986.s001.docx]

**Identification of studies via databases and registers**

Duplicate records removed

(n = 1749)

Records identified from:

Embase (n = 298)

MEDLINE (n= 171)

Scopus (n= 2415)

Pubmud (n= 253)

Web of Science (n = 135)

**Identification**

**Screening**

Records screened by title and abstract

(n = 3353)

Records excluded based on title and abstract

(n = 3011)

Reports excluded:

Non-randomized controlled trial(n = 32)

Unnatural scene (n = 195)

Non-immersive VR approach (n = 61)

Unrelated to the intervention setting(n = 24)

Reports assessed for eligibility

(n = 342)

**Included**

Studies included in review

(n = 30)

Studies of included meta-analysis

(n = 15)

*From:*  Page MJ, McKenzie JE, Bossuyt PM, Boutron I, Hoffmann TC, Mulrow CD, et al. The PRISMA 2020 statement: an updated guideline for reporting systematic reviews. BMJ 2021;372:n71. doi: 10.1136/bmj.n71
